# State-dependent modulation of stimulus-response relations in cortical networks in vitro

**DOI:** 10.1186/1471-2202-12-S1-O6

**Published:** 2011-07-18

**Authors:** Oliver Weihberger, Ayal Lavi, Samora Okujeni, Uri Ashery, Ulrich Egert

**Affiliations:** 1 Bernstein Center Freiburg, University of Freiburg, Freiburg, 79104, Germany; 2 Faculty of Biology, Neurobiology and Biophysics, University of Freiburg, 79104 Freiburg, Germany; 3 IMTEK – Dept. of Microsystems Engineering, University of Freiburg, Freiburg, 79110, Germany; 4 Deptartment of Neurobiology, Tel Aviv University, Tel Aviv, 69978, Israel

## 

Variable responses of neuronal networks to repeated identical electrical or sensory stimuli reflect the interaction of the stimulus’ response with ongoing activity and its modulation by adaptive mechanisms such as cognitive context, network state, cellular excitability or synaptic transmission capability. To identify the rules that underlie the modulation of stimulus-response relations we set up a state-dependent stimulation paradigm in generic neuronal networks *in vitro*.

Extracellular neuronal activity was recorded and stimulated from rat cortical cell cultures on microelectrode arrays at 60 sites. Spontaneous and evoked network activity was examined under control conditions, under blockage of GABA_A_-receptors as well as under overexpression of the synaptic protein DOC2B [1]. We were interested in the interactions that arise between spontaneous and stimulus-evoked activity dynamics and how these shape and modulate stimulus-response relations.

Spontaneous network activity consisted of recurring periods of globally synchronized burst firing, so-called network bursts. The duration of intervals that preceded network bursts best predicted the length of the following network burst. This supported a process of network depression to a low threshold during bursts followed by subsequent recovery [2]. Facilitation of synaptic transmission by overexpressing DOC2B yielded ~30 % more spikes per network burst. The intervals between bursts increased by ~ 75 %, suggesting interdependence between resource activation and the time it needs for them to be recovered.

Response length and delay depended on the timing of stimulation relative to preceding bursting. Response length increased exponentially and saturated with increasing duration of pre-stimulus inactivity t, y(t) = A(1-e^-αt^). Response delay, in turn, decreased exponentially and saturated at a low level, y(t) = Be^-βt^ + C. The rate constant β describes the coupling between recovery from depression and response delay. We found activity-dependent recovery dynamics with longer spontaneous bursts yielding smaller β and vice versa.

Stimulus-response modulation persisted under the blockage of inhibition, that introduced overall longer responses and shorter delays. Longer network bursts with more spikes occurred less frequently and recovery rates concomitantly decreased. The average firing rate was, however, unchanged, supporting a pool of available resources that is repeatedly used and replenished during and between network bursts, respectively.

## Conclusion

The timing of stimulation relative to spontaneous bursting modulates stimulus-response relations in cortical networks *in vitro* following distinct rules. The interrelation between resource depletion and replenishment determines the temporal evolution of the network’s excitability state. Our findings can be explained by short-term synaptic depression and activity-dependent adaptation of excitability as underlying mechanisms.
